# Unsaturated Polyester Resin Nanocomposites Containing ZnO Modified with Oleic Acid Activated by *N*,*N*′-Carbonyldiimidazole

**DOI:** 10.3390/polym10040362

**Published:** 2018-03-24

**Authors:** Hengzhi Chen, Xiaoxue Tian, Jing Liu

**Affiliations:** School of Chemistry and Chemical Engineering, Chongqing University, Chongqing 400044, China; tianxx0919@163.com (X.T.); jliu@cqu.edu.cn (J.L.)

**Keywords:** nanocomposites, grafted polymers, interface, thermal properties

## Abstract

Hydrophobic zinc oxide (ZnO) nanoparticles were produced through grafting aminopropyltriethoxysilane (APS) and oleic acid (OA), which was activated by *N*,*N*′-carbonyldiimidazole (CDI). The functional group containing ZnO nanoparticles were incorporated into unsaturated polyester (UP) resin, and their dispersibility in the UP matrix and effects on the properties of UP/ZnO nanocomposites were investigated. ZnO nanoparticles modified by APS and OA activated by CDI, (CDI–OA–APS–ZnO), can be homogeneously dispersed as supported by transmission electron microscopy (TEM) investigations and had been encapsulated in the UP resin. CDI–OA–APS–ZnO nanoparticles were embedded in the net structure of the UP composites through chemical bonds between oleic acid, styrene, and polyester resin, which significantly influence the cure reaction of UP resin and the properties of UP composites. Thermogravimetric analysis (TGA) results show that the incorporation of ZnO nanoparticles could improve the thermal stability of UP when thermal cracking temperature exceeds 365 °C. The exothermic peak and the initial temperature of cure reaction of the UP resin decreased with increasing ZnO content. The tensile strength and bending strength of UP/CDI–OA–APS–ZnO nanocomposites increased by 91.4% and 71.3% when 3 wt % CDI–OA–APS–ZnO nanoparticles was added into the composites, respectively, compared with pure UP resin.

## 1. Introduction

Unsaturated polyester resins (UPs) are a type of thermosetting resin that are important for their versatility in properties, flexibility in processing, and low cost [1]. However, because of such shortcomings, such as low strength, poor toughness, and large shrinkage, polyester resins should be improved to promote their performance. Nanoparticles usually have more active surfaces and can easily bond to resins with sufficient strength. Also, scattering inorganic nanoparticles into the polymer is considered an effective measure to promote mechanical properties and heat resistant performance [2,3,4]. As a result, inorganic nanoparticle/polymer composites have received increasing concern and interest in recent years [5,6,7]. ZnO nanoparticles have been widely used in scientific research and practical applications because of their non-toxic nature and ability to block UV radiation. ZnO nanoparticles can be used to protect matrix resins from environmental degradation, and increase the toughness and antibacterial property of materials [8,9,10,11]. Moreover, because of their smaller diameter and relatively lower quantity of addition, ZnO nanoparticles have little influence on the materials’ transparency [12].

ZnO nanoparticles are hydrophilic and highly polar, whereas many common polymers, such as polyester resin and polyolefin are nonpolar and hydrophobic. Consequently, the surface of ZnO nanoparticles is often modified for better compatibility and adhesion with the polymer matrix [13,14,15]. Fatty acid and silane couple agents are common surfactants that are used as surface modifier of inorganic nanoparticles [16,17,18]. Nanocomposites composed of ZnO nanoparticles modified by oleic acid that are added into polyaniline (PANI) matrix showed better thermal stability and homogeneous distribution of zinc oxide in PANI [19]. The influence of different coupling agents on the mechanical properties of composite laminates made from a bio-based epoxy resin matrix and basalt fabric was evaluated by Españ et al. Through the addition of silanes, compatibility between basalt fibers and epoxy resins generally increase, which substantially improved their mechanical properties compared to samples without silane treatment [20].

Tzounis et al. [21] had successfully modified the halloysite nanotubes by sodium dodecyl sulfate (SDS) and block copolymer organic materials via noncovalent bonds. The modifications of nanotubes show their good dispersion in the PS polymer matrix and performance regarding thermal and optical properties. The surface morphology and electrical properties of single fibers chemically grafted with carbon nanotubes are studied and compared to physically adsorbing ones. Homogeneous MWCNT networks (multiwall carbon nanotubes) were achieved by chemically grafting carbon nanotubes to the glass fibers, whereas relatively big areas remained uncovered in the case of non-covalently bonded CNTs. The electrical conductivity of single fibers grafted with MWCNTs was generally more than ten times higher than the physically adsorbing ones [22].

Our previous experience showed that the long-carbon chain steric acid activated by *N*,*N*′-carbonyldiimidazole (CDI), had higher reactivity with aminopropyltriethoxysilane (APS) grafted on the surface of ZnO nanoparticles, resulting in a good dispersibility of ZnO particles in the nanocomposites [23,24,25]. Oleic acid (OA) has carbon double bonds, which can conduct the addition reaction with that of unsaturated polyester (UP) resin and styrene. Firstly, we prepared nano-ZnO particles by coprecipitation, which was modified by APS. Then, APS–ZnO was grafted by OA, which was activated by CDI. Lastly, the modified nano-ZnO nanoparticles were incorporated into the polyester matrix and ZnO/UP composites were characterized and evaluated for their mechanical and thermal properties.

## 2. Materials and Methods 

### 2.1. Materials

*N*,*N*′-carbonyldiimidazole (CDI) and aminopropyltriethoxysilane (APS) purchased from Aldrich Chemicals Co. Ltd. (Chengdu, China) were used as activator and coupling agent, respectively. Toluene, zinc nitrate hexahydrate, sodium hydroxide, cyclohexane, oleic acid (OA), cobalt naphthenate, methyl ethyl ketone peroxide (MEKP) were purchased from Chongqing Chuandong Chemicals Co. Ltd. and used as received.

### 2.2. Preparation and Modification of Nano-ZnO

Zn(NO_3_)_2_ and NaOH solutions were added into a three-necked flask. APS/alcohol solution was subsequently dropped into the flask and the mixture was stirred for 4 h. After filtration and washing, the particles were dried in an oven to obtain APS–ZnO nanoparticles. The APS–ZnO nanoparticles were added into the flask, in which OA–CDI had been made in the toluene solvent. The mixture was separated by suction filtration, and extracted with toluene to remove the organic residues, yielding CDI–OA–APS–ZnO nanoparticles. For comparison, one control experiment was performed without CDI.

### 2.3. Preparation of ZnO/UPR Nanocomposites

The ZnO nanoparticles, with different mass ratios to UP resin (1, 3, 5, and 10 wt %), were added into styrene and dispersed by ultrasonic vibration for 30 min, and then UP and the accelerator, i.e., cobalt naphthenate were slowly introduced, and the mixture was vigorous mixed for 2 h. Once nanoparticles were uniformly dispersed in the UP, the initiator, i.e., methyl ethyl ketone peroxide, was added into the mixture, which was further stirred for a few minutes. The hybrid UP composites were left for 10 min and then spread on a tinplate substrate. After airing 72 h, the UP films were peeled carefully from the substrate.

### 2.4. Characterization

Fourier Transform infrared spectroscopy (FTIR) spectrum of modified nano-ZnO particles were obtained on a Nicolet 5DX 550 II spectrometer (Shimadzu, Japan). Thermogravimetric analysis (TGA) was carried out on a Shimadzu model DTG-60H instrument (Shimadzu, Japan) with a heating rate of 10 K/min in flowing N_2_ from ambient temperature to 800 °C. The morphology of ZnO samples were investigated by scanning electron microscopy from an Oxford FEI Nova 400 electron microscope (Hillsboro, OR, USA). The thickness of samples is about 0.5 mm for the superficial test. The film was cut a small patch (about 2 mm × 2 mm) with a razor blade, and the surface was coated with a thin sputtered gold layer because ZnO particles are not conductive. Differential scanning calorimetry (DSC) was carried out on a Mettler Toledo TGA/DSC1 simultaneous thermal analyzer (Shanghai, China) with a heating rate of 5 K/min in flowing N_2_ from ambient temperature to 200 °C. Mechanical properties of the nanocomposites were determined by a universal testing machine (Electro-mechanical Universal Testing Machines, WDT-W, Jinan, China).

## 3. Process and Mechanism

The nano-ZnO particles were prepared and modified with APS and OA. The modified ZnO nanoparticles were then added into polyester resin and styrene hybrids, where crosslinking reactions may occur. The modification sequence and crosslinking reaction are as follows:

- (I) APS–ZnO, ZnO nanoparticles were functionalized by APS (in the following reaction).
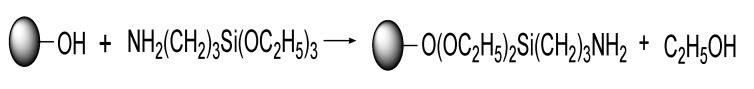
- (II) CDI–OA, OA was activated by CDI (in the following reaction).
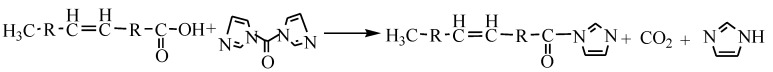
- (III) CDI–OA–APS–ZnO, the condensation reaction between CDI–OA and APS–ZnO was performed (in the following reaction).
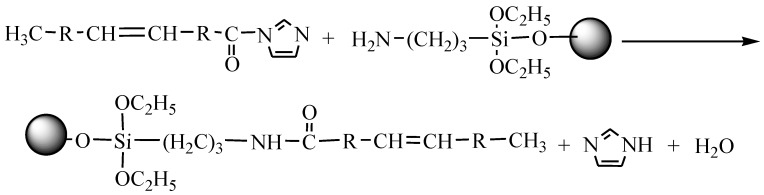
- (IV) APS–ZnO was conducted for comparison (in the following reaction).
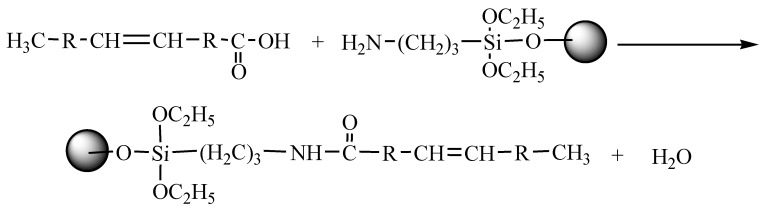
- (V) Preparing CDI–OA–APS–ZnO/UP composites. ZnO nanoparticles were linked on the styrene and polyester resin through addition reaction of carbon double bonds of OA grafted on nano-ZnO (in the following reaction).
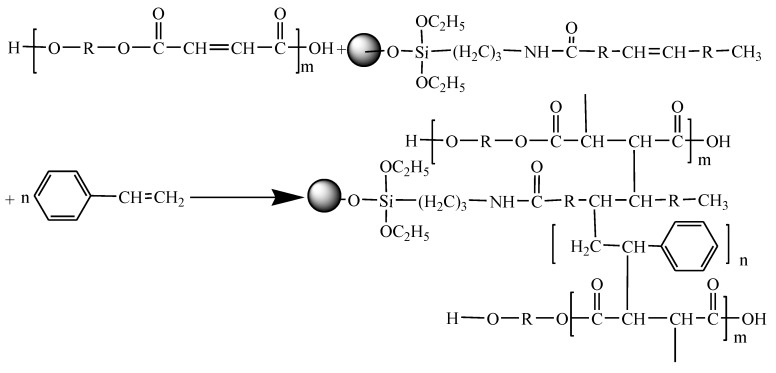

## 4. Results and Discussion

### 4.1. Characteristics of Nano-ZnO Particles

**Figure 1:**
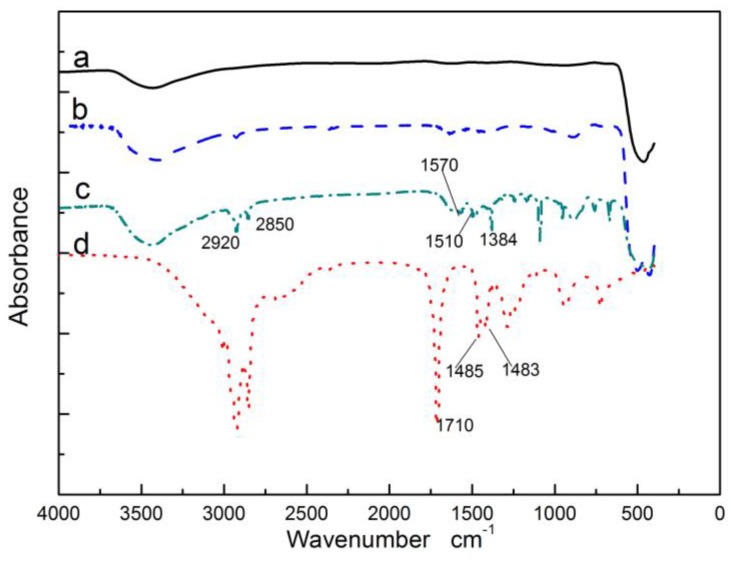
FTIR spectrum of nano-ZnO and the modified nano-ZnO, bare ZnO (**a**), OA–APS–ZnO (**b**), CDI–OA–APS–ZnO (**c**), oleic acid (**d**).

Figure 1 shows the FTIR spectrum of unmodified ZnO (a), OA–APS–ZnO (b), CDI–OA–APS –ZnO (c) and pure OA (d). Compared with spectra a, the peaks at 2920 and 2850 cm^−1^ in spectrum b and c are caused by –CH_2_– stretch vibration. The peak at 1510 cm^−1^ corresponds to the deformation vibration of the NH group of the APS molecule [26], which illuminates that APS was introduced on the surface of the nano-ZnO through chemical bindings, as shown in R1. (Figure 1d) shows the characteristic bands of –COOH and –CH=CH– of OA at 1710 and 1485 cm^−1^, respectively. The disappearance of the peak at 1710 cm^−1^ in spectra d allied with the appearance of the peaks at 1570 and 1433 cm^−1^, which is assignable to C=O and C–O stretching, respectively. This phenomenon confirms the occurrence of the condensation reaction between the carbonyl imidazole and the amino function of APS-ZnO, as shown in R3 [25]. The characteristic band of the –CH=CH– of OA at 1485 cm^−1^ is transferred at 1384 cm^−1^ in the cures of c and d, which indicates that carbon double bonds of OA introduced ZnO nanoparticles were not damaged.

**Figure 2:**
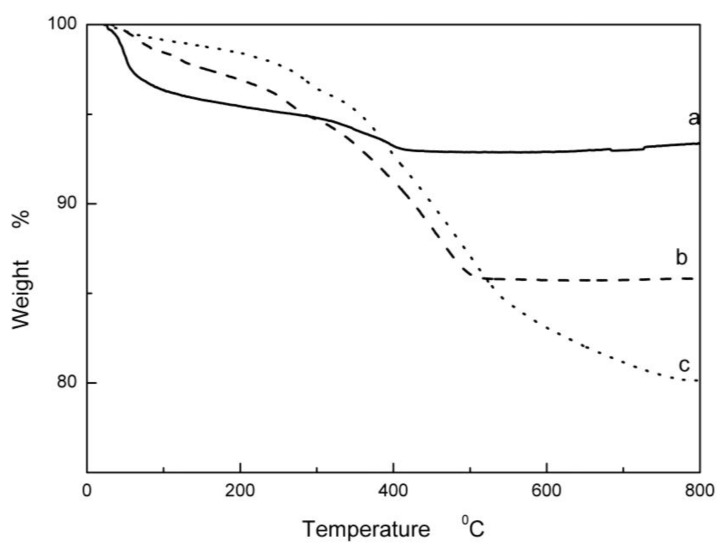
Thermogravimetric analysis (TGA) diagrams of the modified nano-ZnO particles, unmodified ZnO (**a**), OA–APS–ZnO (**b**), CDI–OA–APS–ZnO (**c**).

Figure 2 shows the TG curves of APS–ZnO, OA–APS–ZnO, and CDI–OA–APS–ZnO nanoparticles. It should be noted that the weight ratio of OA/ZnO increases to 25% for investigating the effect of the activator. The ratio of the weight loss of APS–ZnO is 7.2%. The ratios of the weight loss of SA–APS–ZnO particles and CDI–SA–APS–ZnO are 14.2% and 19.9%, respectively, indicating that the amount of OA grafted on the ZnO nanoparticles with CDI is more than that without CDI. The activity of carboxyl groups of OA may not be high enough to give stable covalent bond bindings with the amino groups of APS–ZnO. By contrast, the carboxyl groups of the carbonyl imidazole, an intermediate produced from CDI and OA, has high reaction activation, resulting in the accelerated reaction between the carbonyl imidazole and the amino function of APS–ZnO.

**Figure 3:**
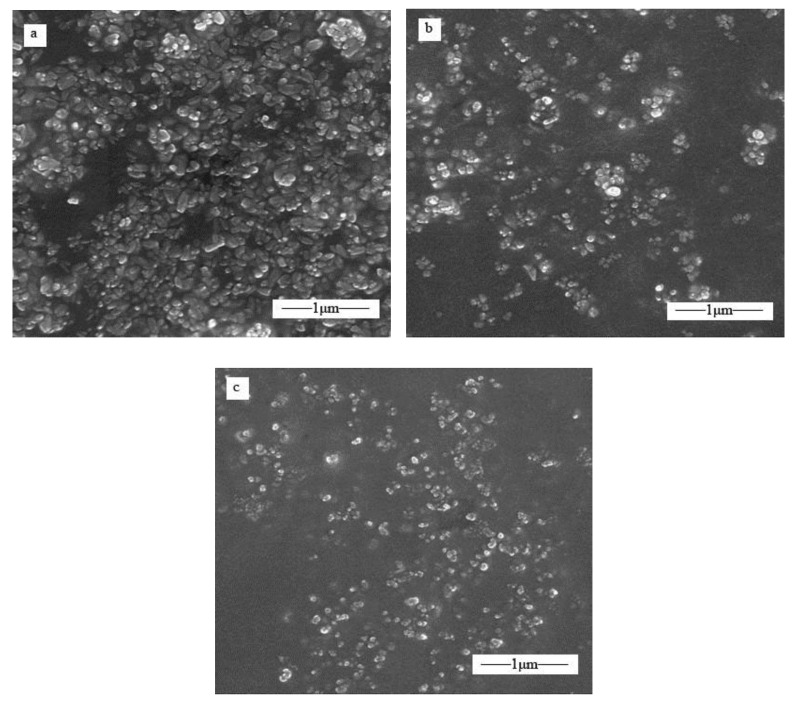
SEM images of nano-ZnO particles, unmodified ZnO (**a**), OA–APS–ZnO (**b**), and CDI–OA–APS–ZnO (**c**).

Figure 3 show the typical SEM images of unmodified ZnO (a), OA–APS–ZnO (b), and CDI–OA–APS–ZnO (c). Clearly, morphological differences may be observed among the three kinds of samples. The individual particle cannot be distinguished due to serious aggregation (Figure 3a). Aggregates of ZnO nanoparticles modified by OA and APS are obviously reduced (Figure 3b). The decreased aggregation of CDI–SA–APS–ZnO particles is evident owing to the sharp decrease in the surface energy caused by the higher amount of OA bonded on the particles through the condensation reaction.

### 4.2. Characteristics of UPR/ZnO Nanocomposites

**Figure 4:**
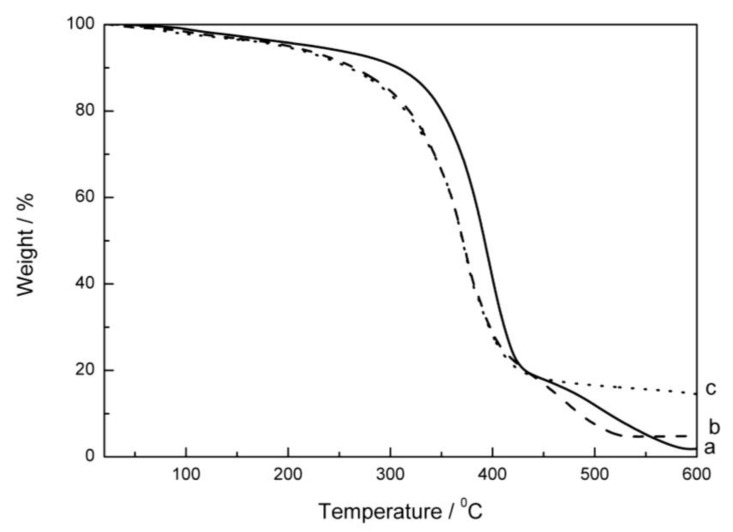
TGA curves of pure unsaturated polyester (UP) (**a**), OA–APS–ZnO/UP (**b**), and CDI–OA–APS–ZnO/UP composites (**c**).

**Figure 5:**
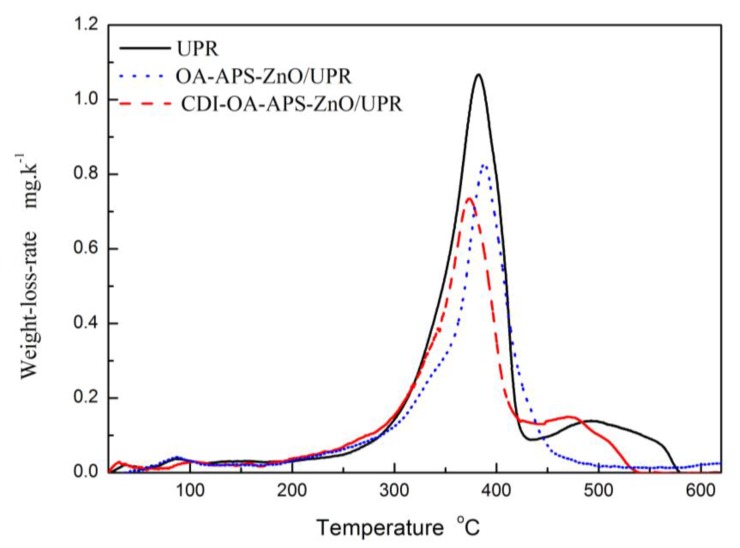
Loss-weight rate of pure UP, OA–APS–ZnO/UP and CDI–OA–APS–ZnO/UP.

The thermal behavior of the UP composite films was investigated using a Shimadzu model DTG-60H instrument (Shimadzu, Japan) (Figure 4). The 10% weight loss observed at 200 °C in the TG curves of pure UP and ZnO/UP composites is attributed to the elimination of the unremoved water molecules in the pure polymers and ZnO/UP composites. Most weight losses occurred between 250 and 500 °C. The ZnO/UP nanocomposites show no significant improvement in thermal stability as compared with pure UP, up to a temperature of 365 °C. After 365 °C, the TG curve of CDI–OA–APS–ZnO/UP composites shifted to higher temperature as compared with pure UP. Thermograms of pure UP resin, OA–APS–ZnO/UP, and CDI–OA–APS–ZnO/UP composites at 600 °C indicate 97%, 92%, and 83% weight losses, respectively. This is probably due to the oleic acid grafted on ZnO. As temperature increases to 365 °C, the oleic acid used as a modifier in UP nanocomposites starts to fragment into small chains, maintaining thermal stability up to a temperature of 365 °C. However, once the chain breaks into small pieces after 365 °C, an increase in thermal stability occurs [17,27]. As shown in Figure 5, weight loss rate of pure UP is clearly greater than that of nano-ZnO/UP composites in the range of 300–420 °C,with the peak values at 1.06, 0.82, 0.75 mg/K for pure UP, OA–APS–ZnO/UP, and CDI–OA–APS–ZnO/UP composites, respectively. These results demonstrate that the thermal stability of the nano-ZnO/UP composites is improved significantly. Comparing to the pure UP, the maximal weight-loss rate of OA–APS–ZnO/UP and CDI–OA–APS–ZnO/UP nanocomposites are cut down 56% and 50.3%, respectively. Similar results were obtained by Kuan et al. [28].

**Figure 6:**
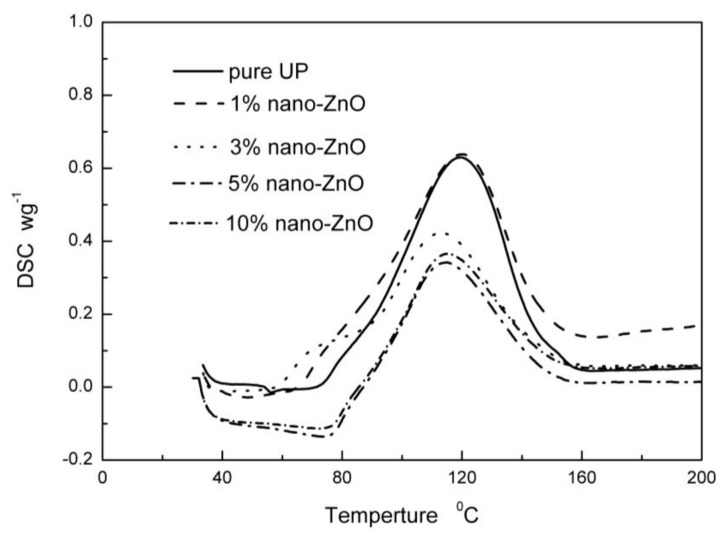
DSC analysis diagrams of the curing reaction process of ZnO/UP nanocomposites.

DSC curves of pure UP and CDI–OA–APS–ZnO/UP nanocomposites are shown in Figure 6. Cure behaviors of pure UP and CDI–OA–APS–ZnO/UP nanocomposites are obviously different. UP and CDI–OA–APS–ZnO/UP nanocomposites start the cure reaction at 83.2 and 80.8 °C. The peak temperature of cure reaction reaches 119 °C for pure UP, whereas the peak temperature is 119.3, 113, 114, and 110 °C for 1, 3, 5, and 10 wt % CDI–OA–APS–ZnO/UP composites, respectively. The decrease in the cure temperature is caused by the carbon double bonds of oleic acid, which have higher reactivity of crosslink reaction than that of polyester resin. Total enthalpy of CDI–OA–APS–ZnO/UP decreases compared to pure UP. The higher the content of CDI–OA–APS–ZnO, the lower the total enthalpy it is. The reason for this phenomenon is that the cure reaction of UP resin follows a free radical reaction mechanism, which involves initiation, propagation, and termination, while nanoparticles usually have a more active surface and can easily bond to free radical ionic. The acidic sites decompose the peroxyketal initiator by a wasteful ionic mechanism, which results in the lower crosslinking reaction rate and exotherms observed [29]. The similar results were obtained by Tzounis et al. [30]. Tzounis et al. employed cyclic butylene terephthalate oligomers as a low molecular weight additive, and added them into the polycarbonate/MWCNT nanocomposite, which resulted in decreasing the temperature of the processing of polycarbonate (PC) filled with MWCNTCOOH.

**Figure 7:**
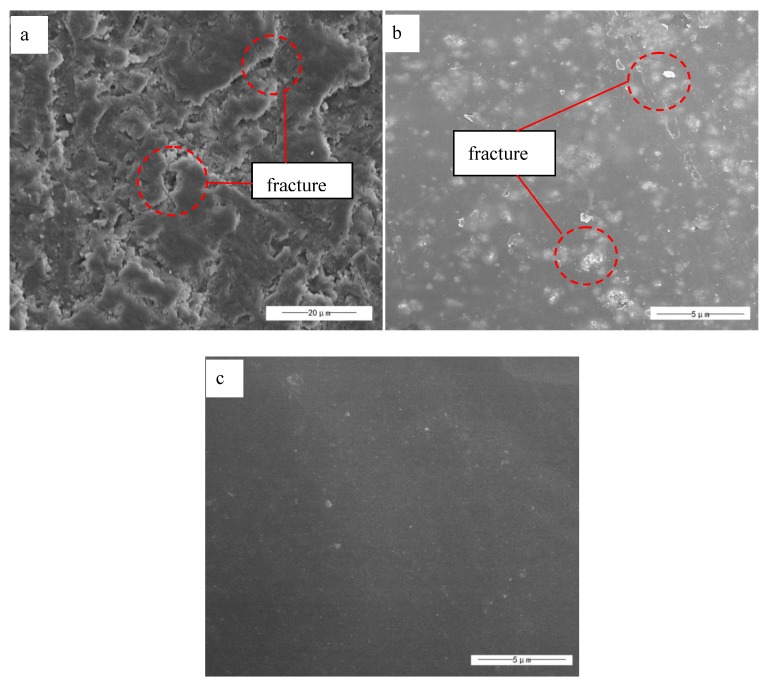
SEM of bare ZnO/UP (**a**), OA–APS–ZnO/UP (**b**), and CDI–OA–APS–ZnO/UP (**c**).

Figure 7 shows the scanning electron micrographs of UP composite films with 3% weight of unmodified ZnO (a), OA–APS–ZnO (b), and CDI–OA–APS–ZnO (c). The unmodified ZnO nanoparticles aggregated heavily, which makes the surface of the unmodified ZnO/UP composite film uneven. Moreover, gaps and chippings caused by the difference in the shrinkage rates between ZnO aggregates and UP resin are clearly observed from the micrograph of the unmodified ZnO/UP composite film. The micrograph of the OA–APS–ZnO/UP composite film became devoid of pinholes and gaps when the dispersibility of OA–APS–ZnO nanoparticles in the UP matrix was improved. However, aggregates were still observed on the micrograph of the OA–APS–ZnO/UP composite film, which may be attributed to the small mass of OA grafted on the ZnO particles. CDI–OA–APS–ZnO nanoparticles were found to be fully integrated into the UP, as seen in the smoother surface of its composite film, compared with that of OA–APS–ZnO/UP (Figure 7c), which implies that a stronger interfacial interaction exists between CDI–OA–APS–ZnO nanoparticles and the UP matrix.

**Figure 8:**
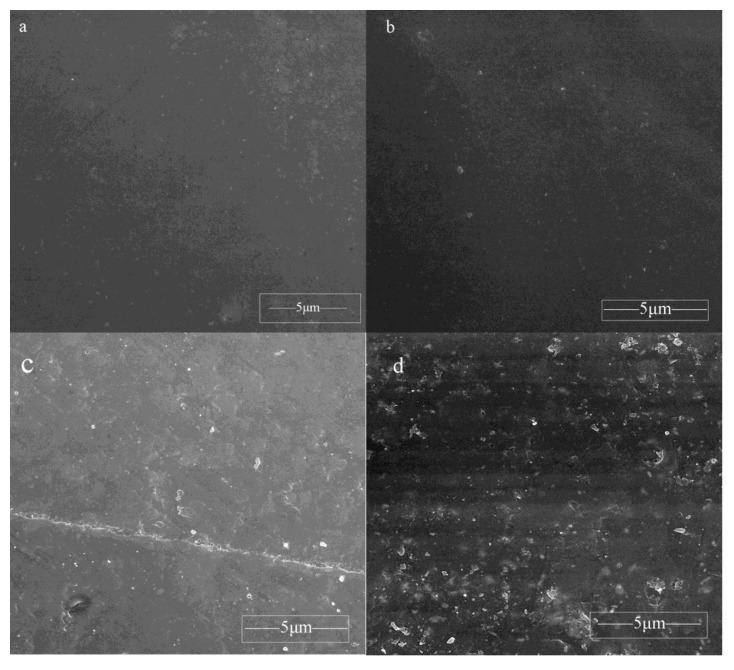
SEM of CDI–OA–APS–ZnO/UP composite containing 1 wt % (**a**), 3 wt % (**b**), 5 wt % (**c**), and 10 wt % (**d**) nanoparticles.

The dispersibility of different additive amount of CDI–OA–APS–ZnO in the UP matrix was also examined as shown in Figure 8. The micrographs depicting the morphology of CDI–OA–APS–ZnO/UP composite films reveal homogeneous and plain structures, and no gap and chipping was observed at 1 and 3 wt % CDI–OA–APS–ZnO. However, the composite films became uneven, and aggregates and chippings were notable when ZnO contents exceeded 3 wt %. This result indicates that an unsuitable additive amount negatively affect the dispersibility of nanoparticles in the UP matrix.

### 4.3. Mechanical Properties

**Figure 9:**
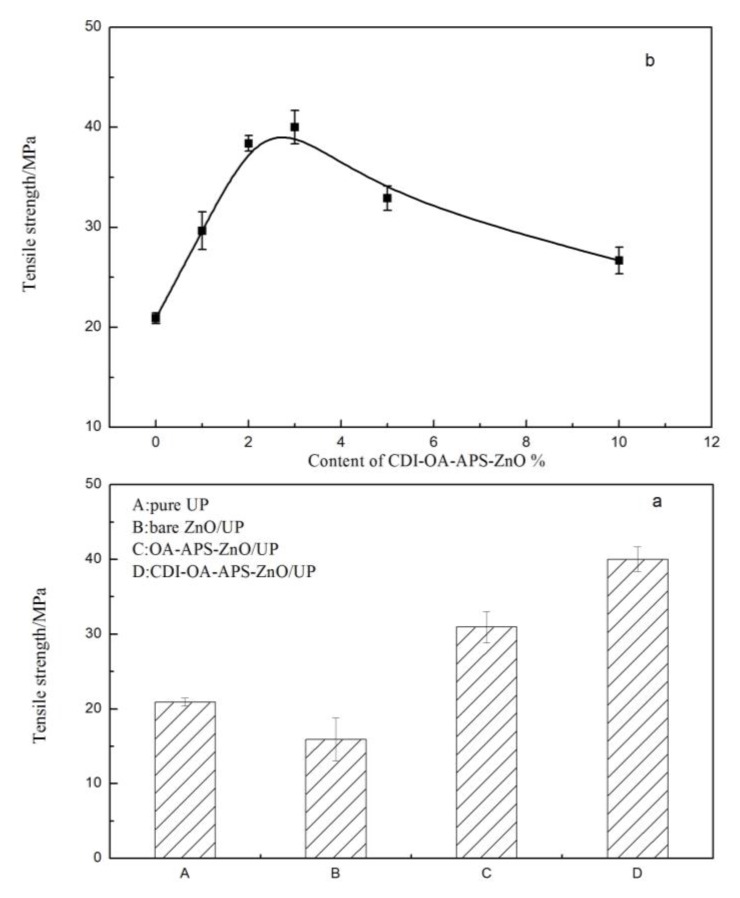
Tensile strength profiles of UP composite films with (**a**) different kinds of ZnO nanoparticles and (**b**) different content.

**Figure 10:**
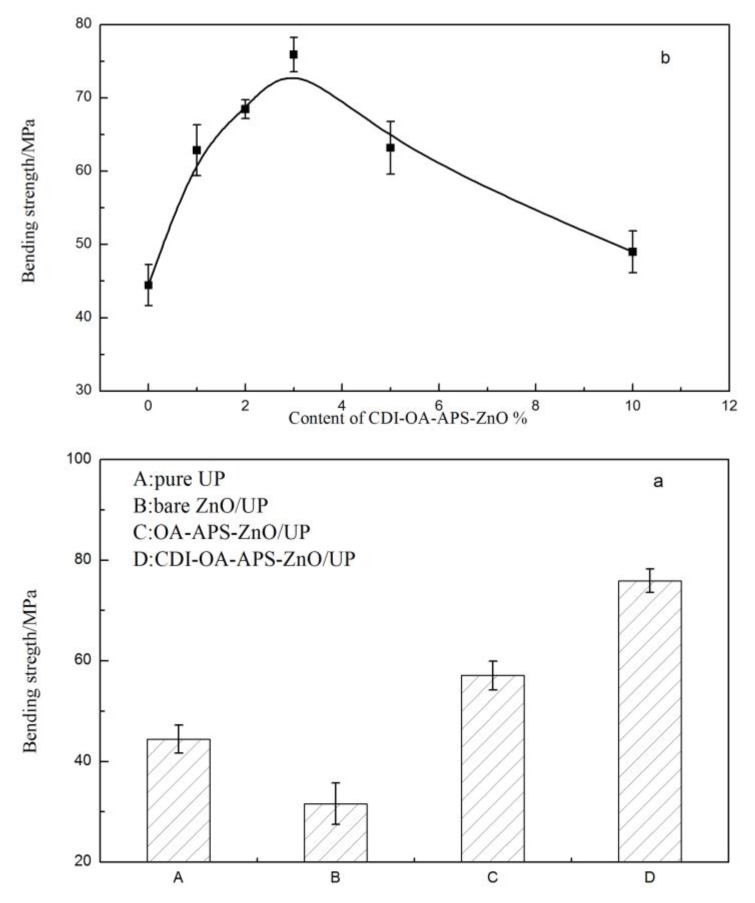
Bending strength profiles of UP composite films with (**a**) different kinds of ZnO nanoparticles and (**b**) different content.

Figure 9a and Figure 10a shows effect of ZnO nanoparticles on the tensile strength and bending strength of pure UP and UP composite films. Tensile strength and bending strength decreased when unmodified ZnO nanoparticles were added into the UP matrix, which contributed to the large surface energy of bare ZnO nanoparticles, resulting in poor dispersibility and serious aggregation in the UP composites. By contrast, the tensile strength and bending strength of the UP composite films containing 3 wt % CDI–OA–APS–ZnO nanoparticles increased by 91.4% and 71.3%, respectively, compared with the pure UP film. This may be attributed to the long carbon chains of OA that effectively entwine with the polymer chain of UP matrix and share some stress [31]. Moreover, the carbon double bonds of OA introduced on the surface of CDI–OA–APS–ZnO nanoparticles can take part in crosslinking reaction with polyester resin and styrene, as shown in R (V). Consequently, the ZnO nanoparticles are embedded in the net structure of the UP composites through chemical bonds, which significantly increase the tensile strength and bending strength of the composites. Figure 9b and Figure 10b show the tensile strength and bending strength for pure UP and UP composites containing various levels of CDI–OA–APS–ZnO nanoparticles. The tensile strength and bending strength of the UP nanocomposite films with the addition of ZnO are greater than that of pure UP film, and the strength of UP composite film reaches up to the maximum at 3 wt % CDI–OA–APS–ZnO particles. At 1 or 3 wt % ZnO particles, the micrographs of PU/CDI–OA–APS–ZnO composite films showed homogeneous and plain structures. However, chippings and gaps were observed in the composite films when ZnO exceeded 3 wt %, leading to a decrease in the tensile strength and bend strength of the nanocomposites.

### 4.4. UV–Vis Spectral Behavior

**Figure 11:**
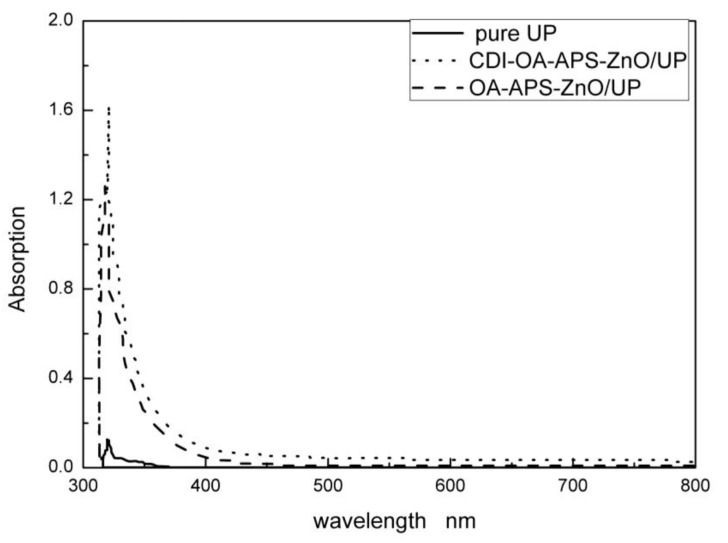
UV–Vis absorbance of ZnO/UP composites.

Figure 11 shows the UV–visible absorbance of pure UP and UP composites with 1 wt % ZnO nanoparticles. Absorbance was remarkably enhanced with the addition of ZnO nanoparticles, compared with pure UP, and the highest intensity was observed in the composite containing CDI–OA–APS–ZnO nanoparticles in the UV range of 300–400 nm. This optical change is caused by the quantum effect of the nanoparticles which is influenced by particle size [32,33]. Few aggregates of CDI–OA–APS–ZnO nanoparticles were observed, which result in a smaller average particles size and higher UV absorbance. Based on the results, CDI–OA–ASP–ZnO/UP composite is a potential UV-shielding material. Also, almost no absorbance in the visible range of 500–800 nm was observed both in pure polymer and the modified ZnO/UP composites, which shows that the addition of ZnO particles has little influence on the transparency of UP resin. 

## 5. Conclusions

ZnO nanoparticles were prepared and modified with OA activated by CDI to improve dispersibility. Results showed that CDI activator promotes the condensation reaction and increases the grafting ratio of OA. The dispersibility of modified ZnO nanoparticle in the UP matrix improved, owing to the enhanced compatibility and adhesion between the UP resin and the modified ZnO particles. The carbon double bonds of OA grafted on nano-ZnO particles can conduct the crosslinking reaction in the composites, thus, the ZnO nanoparticles are connected tightly on the UP resin and styrene through chemical bonds, resulting in the marked influence on mechanical and curing properties of the nanocomposite. The tensile strength and blending strength of UP/CDI–OA–APS–ZnO nanocomposites increased by 91.4% and 71.3% when 3 wt % CDI–OA–APS–ZnO nanoparticles were added into the composites, respectively, compared with pure polyester resin. TGA results show the incorporation of ZnO at temperatures less than 365 °C, while the thermal stability of UP was promoted when the temperature exceeds 365 °C. Compared to the pure UP, the maximal weight-loss rate of OA–APS–ZnO/UP and CDI–OA–APS–ZnO/UP nanocomposites are cut down by 56% and 50.3%, respectively. The cure temperature and exotherm of UP resin decreased with increasing the amounts of nano-ZnO. CDI–OA–ASP–ZnO/UP composite proves to be a potential UV-shielding material with promising versatility owing to the little influence that the addition of ZnO particles have on the transparency of UP resin.
